# Draft Genome Sequences of Four *Streptomyces* Isolates from the *Populus trichocarpa* Root Endosphere and Rhizosphere

**DOI:** 10.1128/genomeA.01344-15

**Published:** 2015-11-12

**Authors:** Dawn M. Klingeman, Sagar Utturkar, Tse-Yuan S. Lu, Christopher W. Schadt, Dale A. Pelletier, Steven D. Brown

**Affiliations:** a Biosciences Division, Oak Ridge National Laboratory, Oak Ridge, Tennessee, USA; b Graduate School of Genome Science and Technology, University of Tennessee, Knoxville, Tennessee, USA

## Abstract

Draft genome sequences for four *Actinobacteria* from the genus *Streptomyces* are presented. *Streptomyces* is a metabolically diverse genus that is abundant in soils and has been reported in association with plants. The strains described in this study were isolated from the *Populus trichocarpa* endosphere and rhizosphere.

## GENOME ANNOUNCEMENT

The *Actinobacteria* phylum contains genera of astonishing diversity of form and function (1). Its members include pathogens of plants, humans and other mammals, nitrogen fixers (*Frankia*)*,* and *Corynebacterium glutamicum* for industrial amino acid production. Genera such as the *Streptomyces* are rich sources for natural products (e.g., antibiotics) and can form endophytic relationships (1–3). *Actinobacteria* are Gram-positive bacteria that usually have high G+C DNA content and are typically filamentous. Our recent bacterial analyses of root microbiomes from mature *Populus deltoides* in Tennessee and North Carolina based on 16S rRNA analyses have identified *Actinobacteria* at the operational taxonomic unit level as, similar to the genus *Streptomyces*, common and often dominant within the endosphere (4). Genome sequences for a number of bacteria isolated from the rhizosphere and endosphere of *P. deltoides* have been reported (5, 6); however, to date, genome sequences for *Streptomyces* isolated from *Populus* have not been reported.

To better understand plant-microbe interactions and to further investigate the role of *Streptomyces* in association with *Populus*, draft genome sequences for three *Streptomyces* strains isolated from the *Populus trichocarpa* endosphere (OK006, OK074, OV320) and one strain from the rhizosphere (OV450) were generated. The strains were isolated from two different common gardens located in Oregon, USA. Strains with the designation OV were collected from Corvallis, and strains designated OK were from Clatskanie.

DNA sequence data were obtained from Illumina paired-end and mate-pair libraries with average insert sizes of 500 bp and 4 kb, respectively; each was sequenced using a MiSeq instrument in 2 × 151-bp configuration according to manufacturer’s protocols. For each genome, multiple assembly approaches were tested as described previously (7), and the SPAdes version 3.1 assembly algorithm (8) gave the best results. Draft genome sequences for strains OV320, OK006, OK074, and OV450, comprised 38, 291, 318, and 390 contigs (>500 bp), respectively. The estimated genome sizes ranged from approximately 9.2 to 11.9 Mb, and the *N*_50_ statistics ranged from 50 to 627 kb. Gene predictions were based on the Prodigal algorithm (9), and the annotations were performed as part of an ORNL annotation pipeline, used previously (7). An analysis of the 16S rDNA sequences revealed that strains OK006, OK074, OV320, and OV450 were 99% identical to cognate sequences from *Streptomyces* sp. JL164 (accession no. AF101414), *Streptomyces lincolnensis* strain cfcc3132 (accession no. GQ258686), *Streptomyces rishiriensis* strain 1706 (accession no. KC336416), and *Streptomyces* sp. 10-1 (accession no. AB222068), respectively. The 16S rDNA sequences from strains OK006, OV320, and OV450 were 97% identical to the 16S rDNA sequence from the model *Streptomyces coelicolor* A3(2), and strain OK074 was 96% identical.

The new *Streptomyces* spp. genomes all encode for cytochrome P450s, which are important enzymes involved in drug metabolism and bioactivation (10), secondary metabolism genes (e.g., polyketide synthases), mono- and dioxygenases, and genes for the breakdown of various cell wall components (e.g., chitinases, endo-1,4-beta-xylanases, endo-1,4-beta-glucanases). The availability of these new bacterial genome sequences, along with those reported previously, and the plethora of genome sequences from ongoing studies will facilitate a deeper understanding of plant-microbe interactions and insights into the *Streptomyces.*

### Nucleotide sequence accession numbers.

This whole-genome shotgun project has been deposited at DDBJ/EMBL/GenBank under the accession numbers LJCU00000000, LJCV00000000, LJCW00000000, and LJCX00000000 for strains OK006, OK074, OV450, and OV320, respectively. The versions described in this report are the first versions, LJCU01000000, LJCV01000000, LJCW01000000, and LJCX01000000.
